# Knowledge, attitudes, and practices among health care providers regarding complementary and alternative medicine in Trinidad and Tobago

**DOI:** 10.1186/s12906-017-1654-y

**Published:** 2017-03-08

**Authors:** Mandreker Bahall, George Legall

**Affiliations:** 1 School of Medicine and Arthur Lok Jack Graduate School of Business, University of the West Indies, St. Augustine, Trinidad and Tobago; 2 University of the West Indies, St. Augustine, Trinidad and Tobago; 3 House #57 LP 62, Calcutta Road Number 3, McBean, Couva, Trinidad, Trinidad and Tobago

**Keywords:** Complementary and alternative medicine, Expectations, Health care provider, Predictors, Satisfaction

## Abstract

**Background:**

Health care providers are often ill prepared to interact about or make acceptable conclusions on complementary and alternative medicine (CAM) despite its widespread use. We explored the knowledge, attitudes, and practices of health care providers regarding CAM.

**Methods:**

This cross-sectional study was conducted between March 1 and July 31, 2015 among health care providers working mainly in the public sector in Trinidad and Tobago. A 34-item questionnaire was distributed and used for data collection. Questionnaire data were analysed using inferential and binary logistic regression models.

**Results:**

Response rate was 60.3% (362/600). Responders were 172 nurses, 77 doctors, 30 pharmacists, and 83 other health care providers of unnamed categories (mainly nursing assistants). Responders were predominantly female (69.1%), Indo-Trinidadian (55.8%), Christian (47.5%), self-claimed “very religious” (48.3%), and had <5 years of working experience (40.6%). The prevalence of CAM use was 92.4% for nurses, 64.9% for doctors, 83.3% for pharmacists, and 77.1% for other health care providers. The majority (50–75%) reported fair knowledge of herbal, spiritual, alternative, and physical types of CAM, but had no knowledge of energy therapy and therapeutic methods. Sex, ethnicity, and type of health care provider were associated with both personal use and recommendation for the use of CAM. Predictors of CAM use were sex, religion, and type of health care provider; predictors of recommendation for the use of CAM were sex and type of health care provider. About half of health care providers (51.4%) and doctors (52%) were likely to ask their patients about CAM and <15% were likely to refer patients to a CAM practitioner. However, health care providers expressed interest in being educated on the subject. Doctors (51.9%) and pharmacists (63.3%) said that combination therapy is superior to conventional medicine alone. Less than 10% said conventional medicine should be used alone.

**Conclusion:**

Knowledge about CAM is low among health care providers. The majority engages in using CAM but is reluctant to recommend it. Predictors of CAM use were sex, religion, and profession; predictors of recommendation for the use of CAM were sex and profession. Health care providers feel the future lies in integrative medicine.

## Background

Advances in conventional health care have led to improved morbidity, mortality, and quality of life. However, complementary and alternative medicine (CAM) is still widely used across the globe. CAM is defined as “a group of diverse medical and health care systems, practices, and products that are not generally considered part of conventional medicine” [1]. The global prevalence of CAM use is 9.8–76.0% [2] and varies greatly from country to country, e.g., 38% in the United States among adults [3], 51.8% in the United Kingdom [4], 68.9% in Australia [5], and 74.8% in South Korea [6]. In Trinidad and Tobago, inhabitants have been traditionally exposed to home remedies and unconventional medical practices [7] that are still used in part due to the lack of available conventional health care [8] but also to improve holistic care [9, 10]. The types of CAM used in Trinidad and Tobago include herbal medicines [11–13], massage [14], Traditional medicine [15], megavitamins, folk remedies, energy healing, and homeopathy [16], which are similar to those used commonly in the United States [17]. Other types of CAM used in Trinidad and Tobago are special prayers [18], a multitude of vitamins for healing and vitality (“boosters”), chelation therapy, Reiki therapy [19], Chinese and Ayurvedic medicine [18] and acupuncture [20].

CAM is of medical interest because of its perceived benefits [10]. According to patients, CAM is used for curing [21], counteracting the side effects of conventional medicine [22, 23], providing and promoting wellness and holistic care [24], maintaining wellness [25], and fulfilling the expectations of health care providers [26]. Many patients present to health care providers to treat complications of CAM [27], including drug interactions [28], and for advice. This is compounded by pharmacists’ and physicians’ lack of knowledge, confidence, and training to provide proper guidance to the increasing number of CAM users [29]. HCPs have also developed a heightened interest about CAM [30]. Furthermore, the issues of safety and efficacy, lack of supporting scientific evidence, and non-disclosure of information [31] have made CAM a major public health problem leading to delayed treatment, disease complications, and even death [32]. This study explores the knowledge, attitudes, and practices of HCPs with regard to CAM.

## Methods

This cross-sectional study was conducted between March 1, 2015 and July 31, 2015 among all doctors, nurses, pharmacists and other clinical staff of any ethnicity and gender working mainly in the public health sector of one of the five Regional Health Authorities in Trinidad and Tobago and general practitioners working in Trinidad. The inclusion criterion was consent to participate in the study. The data collection instrument was a 34-item self-administered questionnaire that included items related to demographics, personal and recommended use of various CAM therapies; knowledge, referral and recommendation, reasons and influences for prescribing CAM; attitudes and practices towards CAM usage in the present and future. The key demographic variables were sex, marital status, ethnicity, educational level, employment status, religion, religiosity, and type of HCP. The sample size of 600 was determined by methods described by Lwanga et al. and represents the minimum sample size required to estimate the percentage of target population who uses CAM with a 4% margin of error [33].

Descriptive and inferential statistical analysis was performed using SPSS version 20 [34] (Chicago, IL, USA). Descriptive methods included frequency tables and graphs for data presentation. Inferential methods included regression analysis and tests of hypothesis including tests of association. Binary logistic regression was used to identify predictors of personal use and recommendation for the use of CAM. All hypotheses were tested at the 5% level of significance.

## Results

A total of 600 questionnaires were distributed, and 362 were returned. The overall response rate was 60.3% (Table 1). Responders were predominantly female (*n* = 250; 69.1%), Indo-Trinidadian (*n* = 202; 55.8%), Christian religious affiliation (*n* = 172, 47.5%), self-claimed “very religious (*n* = 175, 48.3%) and had been in practice for less than five years (*n* = 148; 40.6%).

**Table 1:** Frequency distribution of selected demographics per type of health care provider^a^

Variable	Type of health care provider				
	Doctor *n* (%)	Nurse	Pharmacist	Other	Total
Response rate	77 (21.3)	172 (47.5)	30 (8.3)	83 (22.9)	362 (60.3)
Gender					
Male	45 (58.4)	10 (5.8)	12 (40.0)	45 (54.2)	112 (30.9)
Female	32 (41.6)	162 (94.2)	18 (60.0)	38 (45.8)	250 (69.1)
Years of practice					
< 5	43 (55.8)	49 (25.8)	20 (66.7)	35 (42.7)	148 (40.6)
5–10	8 (10.4)	46 (26.7)	3 (10.0)	10 (12.2)	67 (18.5)
11–20	4 (5.2)	20 (11.6)	4 (13.3)	9 (11.0)	37 (10.2)
21–30	7 (9.1)	16 (9.3)	2 (6.7)	8 (9.8)	33 (9.1)
31–40	13 (16.9)	28 (16.3)	0 (0.0)	13 (15.9)	54 (14.9)
> 40	2 (2.6)	13 (7.6)	1 (3.3)	7 (8.5)	23 (6.4)
Ethnicity					
Afro-Trinbagonian	11 (14.3)	63 (36.8)	1 (3.3)	8 (9.6)	83 (22.9)
Indo-Trinbagonian	50 (64.9)	72 (42.1)	28 (80.0)	56 (67.5)	202 (55.8)
Mixed	11 (14.3)	33 (19.3)	4 (13.3)	14 (16.9)	62 (17.1)
Other	5 (6.5)	3 (1.8)	1 (3.3)	5 (6.0)	15 (4.2)
Religious affiliation					
None	1 (1.6)	10 (7.2)	0 (0.0)	3 (4.4)	14 (3.9)
Islamism	9 (14.8)	8 (5.8)	6 (21.6)	5 (7.4)	28 (7.8)
Hinduism	22 (36.1)	21 (21.0)	7 (30.4)	18 (26.5)	76 (21.0)
Christianity	29 (47.5)	91 (65.9)	10 (43.5)	42 (61.8)	172 (47.5)
Religiosity					
Not religious	12 (16.7)	20 (11.8)	1 (3.3)	9 (10.8)	42 (11.6)
Not very religious	22 (30.6)	59 (34.9)	14 (46.7)	31 (37.3)	126 (34.8)
Very religious	33 (45.8)	87 (51.5)	15 (50.0)	40 (48.2)	175 (48.3)
Extremely religious	5 (6.9)	3 (1.8)	0 (0.0)	3 (3.6)	11 (3.0)

^a^Excludes non-responses

Data are the number (percentage)

**Fig. 1:**
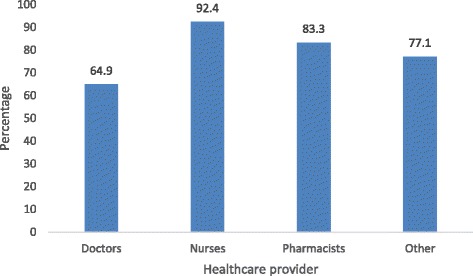
CAM usage per type of health care provider

The prevalence of CAM use was 92.4% (158/172) for nurses, 64.9% (50/77) for doctors, 83.3% (25/30) for pharmacists, and 77.1% (64/83) for other HCPs (Fig. 1). The majority (50–75%) reported fair knowledge of herbal, spiritual, alternative, and physical types of CAM, but no knowledge of energy therapy and therapeutic methods (Table 2). Sex, ethnicity, and profession (type of HCP) were associated with both personal use and recommendation for the use of CAM, while religion, religiosity, and years of practice were not associated (Table 3). Binary logistic regression analysis identified sex, religion, and type of HCP as predictors of CAM use (Table 4), and sex and type of HCP as predictors of recommendation for the use of CAM (Table 5). There was no significant correlation between the reported level of knowledge of CAM and the HCP category, with the exception of pharmacists (Table 6).

**Table 2:** Reported level of knowledge of locally used CAM methods per type of health care provider

			CAM method					
Health care provider	Knowledge level	Herbal *n* (%)		Spiritual *n* (%)	Alternative *n* (%)	Physical *n* (%)	Energy *n* (%)	Therapeutic *n* (%)
Doctors (*n* = 77)	None	4 (5.2)		18 (23.4)	34 (44.2)	14 (18.2)	53 (68.8)	56 (72.7)
	Fair	59 (76.6)		50 (64.9)	33 (42.9)	49 (63.6)	17 (22.1)	16 (20.8)
	Adequate	14 (18.2)		9 (11.7)	10 (13.0)	14 (18.2)	7 (9.1)	5 (6.5)
Nurses (*n* = 172)	None	10 (5.8)		25 (15.1)	72 (41.9)	33 (19.2)	111 (64.5)	107 (62.2)
	Fair	87 (50.6)		113 (65.7)	84 (48.8)	93 (54.1)	50 (29.1)	56 (32.6)
	Adequate	75 (43.6)		33 (19.2)	16 (9.3)	46 (26.7)	11 (6.4)	9 (5.2)
Pharmacists (*n* = 30)	None	0 (0.0)		9 (30.0)	7 (23.3)	5 (16.7)	22 (73.3)	20 (66.7)
	Fair	16 (53.3)		19 (63.3)	21 (70.0)	22 (73.3)	8 (26.7)	10 (33.3)
	Adequate	14 (46.7)		2 (6.7)	2 (6.7)	3 (10.0)	0 (0.0)	0 (0.0)
Others (*n* = 83)	None	3 (3.6)		18 (21.7)	35 (42.2)	19 (22.9)	55 (66.3)	58 (69.9)
	Fair	58 (69.9)		51 (61.4)	41 (49.4)	46 (55.4)	21 (25.3)	20 (24.1)
	Adequate	22 (26.5)		14 (16.9)	7 (8.4)	18 (21.7)	7 (8.4)	5 (6.0)

*CAM* complementary and alternative medicine

Data are the number (percentage)

**Table 3:** Results of chi-square tests of association with personal use of CAM and recommendation for the use of CAM

	Personal use of CAM			Recommendation for the use of CAM		
Variable	*χ* ^2^	df	*p*	*χ* ^2^	df	*p*
Sex	22.16	1	≤0.001	23.81	1	≤0.001
Ethnicity	7.42	2	0.024	6.74	2	0.024
Religion	15.16	4	0.004	4.619	4	0.329
Religiosity	5.5	3	0.138	1.5	3	0.681
Type of health care provider	19.07	3	≤0.001	24.91	3	≤0.001
Years of practice	10.72	5	0.057	6.54	5	0.257

*CAM* complementary and alternative medicine

**Table 4:** Binary logistic regression analysis of personal use of CAM

			95% CI	
	OR	*p*	Lower	Upper
Years of practice	0.927	0.414	0.772	1.113
Sex	2.452	0.010	1.245	4.830
Ethnicity	1.231	0.087	0.970	1.562
Religion	1.351	0.029	1.032	1.769
Religiosity	1.276	0.188	0.887	1.835
Type of health care provider	0.854	0.015	0.753	0.970

*CAM* complementary and alternative medicine, *CI* confidence interval, *OR* odds ratio

**Table 5:** Binary logistic regression analysis of recommendation for the use of CAM

			95% CI	
	OR	*p*	Lower	Upper
Years of practice	0.889	0.340	0.669	1.132
Sex	4.229	0.003	1.649	10.848
Ethnicity	0.988	0.936	0.744	1.314
Religion	1.259	0.216	0.874	1.813
Religiosity	1.004	0.988	0.616	1.636
Type of health care provider	0.854	0.023	0.746	0.978

*CAM* complementary and alternative medicine, *CI* confidence interval, *OR* odds ratio

**Table 6:** Correlation between reported knowledge of CAM and knowledge of specific CAM therapies/methods)

Health care provider	CAM method					
	Herbal r (*p*)	Spiritual r (*p*)	Alternative r (*p*)	Physical r (*p*)	Energy r (*p*)	Therapeutic r (*p*)
Doctors (*n* = 77)	0.481 (≤0.001)	0.587 (≤0.001)	0.618 (≤0.001)	0.518 (≤0.001)	0.482 (≤0.001)	0.507 (≤0.001)
Nurses (*n* = 172)	0.199 (≤0.001)	0.181 (0.291)	0.244 (≤0.001)	0.180 (0.018)	0.215 (0.008)	0.195 (0.010)
Pharmacists (*n* = 30)	0.129 (0.496)	0.163 (0.389)	0.247 (0.188)	0.345 (0.062)	0.179 (0.343)	0.174 (0.359)
Others (*n* = 83)	0.424 (≤0.001)	0.385 (≤0.001)	0.359 (0.001)	0.392 (≤0.001)	0.431 (≤0.001)	0.451 (≤0.001)

*CAM* complementary and alternative medicine

**Fig. 2:**
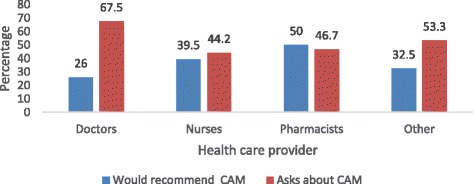
Percentage of health care providers asking patients about CAM usage and who would recommend the use of CAM

**Fig. 3:**
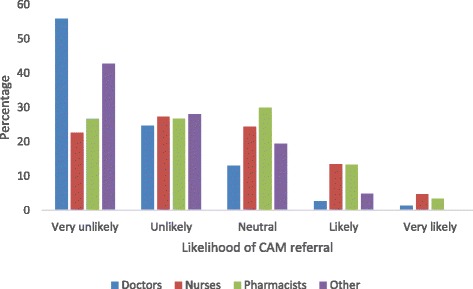
Likelihood of referring a patient to a CAM practitioner by type of health care provider

In general, HCPs remained neutral or were unlikely or very unlikely to recommend CAM. Doctors, more than other HCPs, were most likely to ask their patients about CAM usage (67.5%) and recommend CAM the least (26%). On the other hand, a greater proportion of pharmacists initiated discussion on CAM (46.7%) and recommended CAM (50.0%) (Fig. 2). A small percentage of nurses and pharmacists (<10%) were more likely to refer patients to a CAM practitioner (Fig. 3). When confronted by CAM users regarding side effects or experiences about CAM, most HCPs (>50%) remained neutral and did not offer an opinion on CAM use (Table 7).

**Table 7:** Attitudes of health care providers towards CAM users: *n* (%)

	Ignored patient	Recommended CAM	Remained neutral	Prescribed CAM and CM	Other	No response
Doctors (*n* = 77)	1 (1.3)	15 (19.5)	39 (50.6)	8 (10.4)	9 (11.7)	5 (6.5)
Nurses (*n* = 172)	3 (1.5)	8 (8.2)	90 (52.6)	22 (12.9)	18 (10.5)	24 (14.0)
Pharmacists (*n* = 30)	0 (0.0)	2 (6.7)	11 (36.7)	9 (30.0)	5 (16.7)	3 (10.0)
Others (*n* = 83)	3 (3.6)	12 (14.5)	40 (48.2)	9 (10.8)	13 (15.7)	6 (7.2)

*CAM* complementary and alternative medicine, *CM* conventional medicine

Data are the number (percentage)

Doctors (51.9%), nurses (43.0%), pharmacists (63.3%), and other HCPs (43.4%) said that combination therapy is superior to Conventional Medicine alone. HCPs believed that combination therapy increases patient satisfaction and assists in fighting illness (Table 8). The superiority of CAM, including greater satisfaction, ability to fight illness, and promotion of health and wellbeing, was supported mainly by pharmacists. HCPs, particularly doctors and nurses, feel the future lies in integrative medicine combining conventional medicine and evidence-based CAM (Table 9). Less than 10% of HCPs said conventional medicine should be used alone, and 12–26% said conventional medicine and CAM should be used at the discretion of the HCP.

**Table 8:** Attitudes towards CAM: *n* (%)

	Doctors (*n* = 77)	Nurses (*n* = 172)	Pharmacists (*n* = 30)	Others (*n* = 83)
Practicing with knowledge of CAM and CM is superior to practicing with only knowledge of CM	40 (51.9)	74 (30.0)	19 (63.3)	36 (43.4)
Incorporation of CAM therapies can result in increased patient satisfaction	43 (55.8)	100 (58.1)	21 (70.0)	53 (63.9)
CAM therapies can assist in fighting illness	40 (51.9)	103 (59.9)	19 (63.3)	49 (59.0)
CAM therapies can promote general health and wellness	48 (62.3)	110 (64.0)	21 (70.0)	53 (63.9)
Medical practitioners should be more educated in the use of CAM	65 (84.4)	43 (83.1)	25 (83.3)	63 (75.9)
Would support incorporation of CAM in the medical curriculum	53 (68.8)	126 (73.3	23 (76.7)	55 (63.3)
Incorporation of CAM therapies into the into the health care system would enhance patient care	46 (59.1)	126 (73.3)	18 (60.0)	52 (62.7)
Would support CAM being introduced in a drug formulary	34 (44.2)	117 (60.8)	16 (53.3)	38 (45.8)
Research on the efficacy and safety of CAM should be performed	63 (81.8)	142 (82.6)	25 (83.3)	66 (75.9)
Provision of wellness centres using CAM and CM would benefit patients	48 (62.3)	132 (76.7)	22 (73.3)	61 (74.4)

*CAM* complementary and alternative medicine, *CM* conventional medicine

Data are the number (percentage)

**Table 9:** How respondents see the future of CAM in medical practice: *n* (%)

	Doctors (*n* = 77)	Nurses (*n* = 172)	Pharmacists (*n* = 30)	Others (*n* = 83)
CM alone	8 (10.4)	6 (3.5)	2 (6.7)	3 (3.6)
CM and CAM at the patient’s discretion	9 (11.7)	40 (23.3)	2 (6.7)	7 (8.4)
CM and CAM at the doctor’s discretion	11 (14.3)	31 (18.0)	8 (26.7)	10 (12.0)
CM and evidence-based CAM as integrative medicine	47 (61.0)	73 (42.4)	16 (53.3)	55 (66.3)
No response	2 (2.6)	22 (12.8)	2 (6.7)	7 (8.4)

*CAM* complementary and alternative medicine, *CM* conventional medicine

Data are the number (percentage)

## Discussion

### Personal usage and prescribing practices

In this study, the overall prevalence of CAM use among HCPs was high (82.3%): nurses (92.4%), pharmacists (83.3%), other HCPs (82.3%), and doctors (64.9%). These are quite high rates considering HCP’s training in evidence-based medicine. However, such prevalence rates are similar to those of other countries with folk medicine tradition: 100% of pharmacy students in Sierra Leone [35] and 51% of physicians at a paediatric hospital in Mexico [36]. In a previous study in Trinidad, 40.6% of physicians admitted to having used herbs [11]. Prevalences in developed countries are also quite high: 36 and 12% among nurses and doctors, respectively, in Norway [37] and 40% [29] and 76% [38] among healthcare workers in two US studies. Predictors of CAM were found to be sex, religion, and HCP category. Spirituality and religiousness were found as predictors in other studies [39].

### CAM recommendation

Despite the high HCP usage (Fig. 2), only 26% of doctors recommended its usage. Wide variations exist between personal use of CAM and CAM recommendation. Similar to our study, Maha and Shaw found that most HCPs who use CAM personally, do not recommend it [40]. Clement et al. found that 40.6% of physicians have used herbs but only 27.1% recommended them to their patients [11]. HCPs, particularly doctors, may feel reluctant to prescribe CAM because of insufficient knowledge and evidence to justify CAM prescription, and ethical and legal obligations to patients. It may be difficult to justify the use of CAM (chelation therapy to dissolve plaques in CAD, boosters to improve strength and vitality, or herbs for cancer treatment) in the presence of well-tested conventional medicine, and when CAM is reported as unsafe and ineffective [41] by some physicians (18.5%). This is in contrast to other studies that found that physicians who used CAM previously are more likely to recommend CAM to their patients [42, 43].

Doctors particularly felt that recommendations should be based on evidence-based guidelines, as in other studies [44, 45]. The perceived safety and its feature as a natural product by pharmacists made them more likely to prescribe CAM [46]. This may contribute to the 50% of pharmacists who recommended CAM in our study.

### Education and knowledge

The majority of doctors (84.4%), nurses (83.1%), and pharmacists (83.3%) felt that medical practitioners should be more educated on CAM. The desire to learn about CAM goes beyond curiosity and information but to acquire the knowledge to treat complications and drug interactions [27] and to communicate effectively with patients about CAM [47]. Physicians were generally more interested in learning about CAM therapies, while nurses regarded it as less important to have knowledge about CAM in comparison to other HCPs. Nonetheless, more than twice as many nurses (43.6%) and pharmacists (46.7%) as doctors (<20%) reported to have adequate knowledge of herbal CAM.

### CAM communication

Our study reveals that when confronted by CAM users on medical issues, the majority of doctors (50.6%) and nurses (52.6%) remain neutral or non-committal. This is despite the benefits and importance of communication between patients and doctors [48]. This may result from their inexperience or incompetence [30, 49] or lack of knowledge as was found in this study. Despite the multiple concerns, few doctors are prepared to listen to patients on CAM usage [48, 50]. Patients are, therefore, left unmonitored by professionals compromising safety and effectiveness of CAM therapies as in Trinidad.

### CAM usage

At least 81.8% of doctors, 82.6% of nurses, and 83.3% of pharmacists felt that CAM should be tried and scientifically tested before usage. A substantial percentage of doctors (44.2%) also felt it should be placed in a drug formulary. Most HCPs (doctors (61%), nurses (42.4%), pharmacists (53.3%), and other HCPs (67.1%)) believe that conventional medicine and evidenced based CAM should be integrated. Less than 35% of HCPs felt that the combination of treatment should be at the doctors or patients’ discretion while a small but significant percentage of doctors (10.4%), nurses (3.5%), pharmacists (6.7%) and other HCPs (3.7%) felt CM should be used alone.

### Future perspectives

The patients’ perceived benefits of CAM in holistic care [51–53], longevity [54], quality of life [10], as well assisting in the counteracting or destroying side effects of CM [22, 55] have encouraged its usage. Though Marusic’s statement, “there is no sound proof of CAM effectiveness, no hypotheses on the mechanisms of their action, nor scientific reports testing them” [56] is contentious, there is a lack of evidence for most CAM. Our study revealed that healthcare providers prefer evidence based guidelines (EBG) of CAM for its recommendation. Controlled studies to determine outcomes such as health-related quality of life and additional outcomes related to whole-person health—physical, mental, social, and spiritual should be encouraged and emphasised [57]. Doctors are also concerned about “safety, lack of proof that therapies work, inadequate knowledge and experience with CAM among doctors and absence of statutory regulation for most therapies” [58, 59], thus making efforts to ensure safety, efficacy, and quality; access; and rational CAM use [60]. Participants of the study believe that the future of therapeutics lies in integrative medicine (Conventional Medicine and evidence-based CAM) as was also pointed out by Dobos [61].

This study has some limitations. First, the sample was not randomized; however, the questionnaires were distributed widely to nurses, doctors, and pharmacists across different departments and locations. Second, the sample was too small for subgroup analysis. Third, answers depended on memory recall, which may introduce bias. The sample, though comprising a group of experts, is influenced both from their heritage of centuries of traditional medicine from fore parents from Africa and India as well as modern day exposure to Chinese, Ayurvedic, and western (USA) and South American medicine. While the findings may be unique to Trinidad, patients’ characteristics and CAM practices are similar to other countries and these findings can be generalised to other HCPs in other countries.

## Conclusions

The prevalence of CAM use among HCPs in Trinidad and Tobago was high (82.3%). CAM use was more prevalent among nurses, followed by pharmacists, doctors, and other HCPs. However, knowledge about CAM was low, particularly among doctors, and the majority was reluctant to recommend CAM and to refer patients to a CAM practitioner. Sex, ethnicity, and type of HCP were associated with both personal use and recommendation for the use of CAM. Predictors of CAM use were sex, religion, and profession; predictors of recommendation for the use of CAM were sex and profession. Pharmacists, followed by doctors, other HCPs, and nurses, feel combination therapy is superior to CM alone. HCPs, particularly doctors and nurses, feel the future lies in integrative medicine combining Conventional Medicine and evidence-based CAM. Only a small percentage of HCPs feel CM should be used alone. There was inadequate communication with HCPs, leaving patients largely unsupervised and unmonitored by medical personnel.

## Abbreviations

CAM: Complementary and alternative medicine
CM: Conventional medicine
HCP: Health care provider
NCCAM: National centre for complementary and alternative medicine
WHO: World Health Organization
